# A Critical Evaluation and Modification of the Padé–Laplace Method for Deconvolution of Viscoelastic Spectra

**DOI:** 10.3390/molecules26164838

**Published:** 2021-08-10

**Authors:** Siamak Shams Es-haghi, Douglas J. Gardner

**Affiliations:** Advanced Structures and Composites Center, The University of Maine, 35 Flagstaff Road, Orono, ME 04469-5793, USA; douglasg@maine.edu

**Keywords:** viscoelasticity, rheology, stress relaxation, Padé approximant, Toeplitz matrix, condition number, ill-conditioned systems

## Abstract

This paper shows that using the Padé–Laplace (PL) method for deconvolution of multi-exponential functions (stress relaxation of polymers) can produce ill-conditioned systems of equations. Analysis of different sets of generated data points from known multi-exponential functions indicates that by increasing the level of Padé approximants, the condition number of a matrix whose entries are coefficients of a Taylor series in the Laplace space grows rapidly. When higher levels of Padé approximants need to be computed to achieve stable modes for separation of exponentials, the problem of generating matrices with large condition numbers becomes more pronounced. The analysis in this paper discusses the origin of ill-posedness of the PL method and it was shown that ill-posedness may be regularized by reconstructing the system of equations and using singular value decomposition (SVD) for computation of the Padé table. Moreover, it is shown that after regularization, the PL method can deconvolute the exponential decays even when the input parameter of the method is out of its optimal range.

## 1. Introduction

The representation of a function as the sum of exponential decays can be observed in many areas of science and engineering such as solid-state physics, chemical kinetics, biology, and rheology of polymers [1]. For example, in relaxation phenomenon of polymers, the experimental data can be mathematically described as a linear combination of multiple exponential functions. The parameters involved in the linear combination of exponential functions (amplitudes and decay constants) have physical significance, and to extract these constants, an inverse problem that is inherently ill-posed [2] must be solved. In the relaxation phenomena of polymers, the decay constants are related to the relaxation times and the amplitudes are the corresponding weights. The set of relaxation times and their corresponding weights represents the discrete relaxation spectrum that is considered as the fingerprint of each polymer and can be used in formulation of constitutive equations and in prediction of rheological properties of polymers such as zero shear viscosity, dynamic moduli, and dynamic viscosities. The relaxation spectra of polymers are related to the dynamics of polymer chains, which highly depends on the molecular characteristics of polymer chains such as their chemical structure, polymer chain architecture, molecular weight, and molecular weight distribution. It is important to note that the number of exponential modes is not known a priori and must be determined. In [1] and references therein, there are several proposed approaches for solving the problem of separation of exponentials. Additionally, a detailed review of some other numerical techniques of exponential analysis can be found in [3]. Among the numerical procedures presented for multi-exponential analysis, the Padé–Laplace (PL) method developed by Yeramian and Claverie [4] can deconvolute exponential decays without using initial guesses for the constants [3]. In addition to that, the number of exponential modes is an outcome of the numerical procedure and only one input parameter is required to extract the exponential modes [4]. The PL method combines the Laplace transform and Padé approximation to address the ill-posed problem of separation of exponentials, and its key step in deconvolution of exponentials is the computation of Padé approximants [4], for which Yeramian and Claverie [4]. used the algorithm proposed by Longman [5]. However, as described by Tang and Norris [6], the Longman algorithm can be unstable, especially when the first few data points are close to zero. In a response to Tang and Norris [6], Yeramian [7] indicated that the Longman method is just an efficient numerical tool that allows one to calculate the coefficients of the Padé approximants. More importantly, Yeramian [7] mentioned that “*To solve the linear system equations corresponding to each Padé approximant we may use simple determinants*”. 

The PL method has been used in deconvolution of the relaxation spectra of polymers [8,9,10,11,12], but the points raised by Tang and Norris [6] regarding the instability of the Longman method and in general the problematic computation of Padé approximants have been, so far, overlooked. Knowing the fact that the computation of Padé approximants is a crucial step in PL numerical procedure, an analysis of the PL method, specifically the computation of Padé approximants and the possibility of ill-posedness caused by this computation, is still lacking. Therefore, the objective of this paper was to analyze the PL method in separating exponential functions with an emphasis on the computation of Padé approximants in PL theory.

The paper is organized as follows: The second section gives a brief overview of the Padé-Laplace theory, discusses the numerical implementation, and presents some indications of the propensity for ill-posedness. The third section analyzes the computation of Padé approximants from discrete data points generated by known multi-exponential functions. In this section, we show that the PL method can create a system of equations with large condition numbers. In section four, a method for regularization of the PL method is used and we demonstrate its success in several examples. The paper closes with the conclusions of the results.

## 2. The Padé-Laplace Theory

This section gives a brief overview of the PL method and presents important issues related to this numerical procedure. The complete details of the PL theory can be found in [4].

As mentioned earlier, the PL method uses a combination of Laplace transform and Padé approximants to extract the number of exponential modes, amplitudes, and decay constants associated with each mode, whose summation results in given experimental data obtained in discrete time intervals. In other words, the experimental data f(t) may be expressed as

(1) $$f\left(t\right)=\overset{n}{\underset{k=1}{\sum }}\alpha_{k}\exp\left(\beta_{k}t\right),$$

where n, $\alpha_{k}$, and $\beta_{k}$ are number of modes, amplitudes, and decay constants of mode k, respectively. In general, the exponents can be complex numbers. 

Applying the Laplace transform to Equation (1) gives

(2) $$F(p)=\overset{n}{\underset{k=1}{\sum }}\frac{\alpha_{k}}{p-\beta_{k}},$$

where Re(p)>supk[Re(βk)] and F(p) is

(3) $$F(p)=\int_{0}^{\infty }e^{-pt}f(t)dt.$$

In Equation (2), amplitudes appear as the residues of F(p) and exponents are the poles. The first step of the PL method is to express F(p) as a polynomial function using a Taylor series expansion about some point $p_{0}$ truncated to some order K such as

(4) $$F(p)≃\overset{K}{\underset{k=0}{\sum }}c_{k}{\left(p-p_{0}\right)}^{k},\;c_{k}=1/k!(d^{k}F(p)/dp^{k})(p_{0}),$$

where $\left(d^{k}F(p)/dp^{k})(p\right)$ is given by

(5) $$\frac{d^{k}F(p)}{dp^{k}}(p)=\int_{0}^{\infty }{\left(-t\right)}^{k}f(t)\exp(-pt)dt.$$

Since the values of f(t) are known at discrete time intervals,$\;c_{k}$ can be calculated by numerical integration. 

The second step of the PL method is to construct the Padé approximant of the polynomial found by the Taylor expansion, Equation (4). In other words, the polynomial function should be expressed by the division of two polynomials as

(6) $$\overset{K}{\underset{k=0}{\sum }}c_{k}{\left(p-p_{0}\right)}^{k}=\frac{\overset{n-1}{\underset{k=0}{\sum }}a_{k}{\left(p-p_{0}\right)}^{k}}{\overset{n}{\underset{k=0}{\sum }}b_{k}{\left(p-p_{0}\right)}^{k}},\;K=2n-1.$$

All the papers that implemented the PL method [4,8,9,10,11,12] considered the condition $b_{0}=1$ for the construction of the Padé approximants, Equation (6), which can result in ill-posedness of the PL method. In effect, as will be demonstrated in this section, after applying $b_{0}=1$, a system of equations will be extracted from Equation (6) that, by performing analysis and numerical computations later in the paper, will show that these equations are ill-conditioned. 

Upon constructing the Padé approximants, $\alpha_{k}$ and $\beta_{k}$ can be extracted by finding the poles and residues of the Padé approximants in a comparison between the right-hand sides of Equations (6) and (2). 

The only input parameter of this method is $p_{0}$, that is, the point over which the Taylor series is expanded. Theoretically, the results of the computation must be independent of $p_{0}$; however, this is not the case in reality. According to previous papers [4,8,9,10,11,12], the problem with some values of $p_{0}$ is attributed to the round-off errors. Therefore, it was suggested that $p_{0}$ must be chosen in an optimal range. Aubard et al. [13] proposed an optimal range as a rule of thumb in the interval between the largest and smallest values of absolute values of $\beta_{k}$, i.e., $\left[\inf_{k}\left|\beta_{k}\right|,\sup_{k}\left|\beta_{k}\right|\right]$. Hereafter, by optimal range for a function, we suggest the optimal range proposed by Aubard et al. [13] As a practical supposition, Hellen suggested that a good choice for $p_{0}$ can be the inverse of the time that it takes for the data points to decay to the half of the initial value [14]. However, later we will show that it is possible to achieve the results even when $p_{0}$ is out of the optimal range. 

After Taylor expansion in Laplace space, by increasing the level of the Padé approximant, poles and residues are calculated at each Padé level and then the stable modes are identified. The stable modes are the modes that appear in Padé table and remain in the subsequent Padé levels. The number of stable modes determines the number of exponential modes. Therefore, the number of modes is an outcome of the PL numerical procedure. 

To construct the Padé approximant from Equation (6), a system of linear equations should be solved. Considering $b_{0}=1$, and expanding the summations in Equation (6), one arrives at

(7) $$\begin{array}{l} c_{0}+c_{1}(p-p_{0})+c_{2}{\left(p-p_{0}\right)}^{2}+\cdots +c_{2n-1}{\left(p-p_{0}\right)}^{2n-1}=\\ \frac{a_{0}+a_{1}(p-p_{0})+a_{2}{\left(p-p_{0}\right)}^{2}+\cdots +a_{n-1}{\left(p-p_{0}\right)}^{n-1}}{1+b_{1}(p-p_{0})+b_{2}{\left(p-p_{0}\right)}^{2}+\cdots +b_{n}{\left(p-p_{0}\right)}^{n}}. \end{array}$$

Multiplying both sides by the denominator of the right-hand side and comparing the terms with identical powers of $\left(p-p_{0}\right)$, we find (see Appendix A)

(8) $$\begin{array}{ll} a_{0}=c_{0},\; & \\ a_{k}=c_{k}+\overset{k}{\underset{i=1}{\sum }}b_{i}c_{k-i}, & 0<k\leq n-1\\ c_{k}+\overset{n}{\underset{i=1}{\sum }}b_{i}c_{k-i}=0, & n\leq k\leq 2n-1 \end{array}$$

The third part of Equation (8) can be expanded as

(9) $$\left(\begin{array}{ccccc} c_{n-1} & c_{n-2} & c_{n-3} & \ldots & c_{0}\\ c_{n} & c_{n-1} & c_{n-2} & \ddots & \vdots \\ c_{n+1} & c_{n} & c_{n-1} & \ddots & c_{n-3}\\ \vdots & \ddots & \ddots & \ddots & c_{n-2}\\ c_{2n-2} & \ldots & c_{n+1} & c_{n} & c_{n-1} \end{array}\right)\left(\begin{array}{c} b_{1}\\ b_{2}\\ \vdots \\ b_{n-1}\\ b_{n} \end{array}\right)=\left(\begin{array}{c} -c_{n}\\ -c_{n+1}\\ \vdots \\ -c_{2n}\\ -c_{2n-1} \end{array}\right),$$

and this system of equations must be solved for b values. After doing so, the $a_{k}$ values will be calculated from the second part of Equation (8). Solving the system of equation given by Equation (9) is the major part of the PL method.

The coefficient matrix in this system of equations, Equation (9), is a Toeplitz matrix whose entries are the coefficients of the Taylor expansion calculated in the Laplace space. In the next section, we will show that the Toeplitz matrices that appear in computation of Padé approximants are close to singular. In effect, it will be shown that for different sets of synthesized data points from known exponential functions the condition numbers of the Toeplitz matrices become quite large, which results in ill-conditioned systems of equations. 

The level of the Padé approximants determines the size of the Toeplitz matrix. For example, when n=3, the Padé approximant, which is shown by [2/3], is

(10) $$[2/3]=\overset{2}{\underset{k=0}{\sum }}a_{k}{\left(p-p_{0}\right)}^{k}/\overset{3}{\underset{k=0}{\sum }}b_{k}{\left(p-p_{0}\right)}^{k},$$

and is related to a 3×3 Toeplitz matrix

(11) $$\left(\begin{array}{ccc} c_{2} & c_{1} & c_{0}\\ c_{3} & c_{2} & c_{1}\\ c_{4} & c_{3} & c_{2} \end{array}\right).$$

Therefore, to achieve higher levels of Padé approximants, the size of the Toeplitz matrix must increase. 

## 3. Ill-Posedness of the PL Method

The condition number of coefficient matrix in a system of linear equations is the most important indicator in analyzing the stability of computations and numerical sensitivity [15]. In Section 2, we explained that to extract the Padé coefficients, a system of linear equations, whose coefficient matrix is a Toeplitz matrix, must be solved. To show the structure of the Toeplitz matrix in the deconvolution process of the sum of exponential functions using the PL method, we consider different sets of data points generated from known exponential functions and use the procedure explained in Section 2 to construct the Toeplitz matrix for each Padé level. For all the calculations in this paper, we used the trapezoidal rule for the numerical integration of Equation (5).

Table 1 shows the Toeplitz matrices for the different Padé levels calculated for a three-component function f(t), which is

(12) $$f\left(t\right)=150e^{-0.004t}+19e^{-0.04t}+30e^{-0.06t}.$$

In Table 1, the Toeplitz matrices corresponding to each Padé level and their 2-norm and infinity-norm condition numbers are given. The coefficient matrices, given by Equation (9), associated with each Padé level in Table 1 are given by

(13) $$[0/1]\to c_{0},\;[1/2]\to \left(\begin{array}{cc} c_{1} & c_{0}\\ \;c_{2} & c_{1} \end{array}\right),\;[2/3]\to \left(\begin{array}{ccc} c_{2} & c_{1} & c_{0}\\ c_{3} & c_{2} & c_{1}\\ c_{4} & c_{3} & c_{2} \end{array}\right),\;[3/4]\to \left(\begin{array}{cccc} c_{3} & c_{2} & c_{1} & c_{0}\\ c_{4} & c_{3} & c_{2} & c_{1}\\ c_{5} & c_{4} & c_{3} & c_{2}\\ c_{6} & c_{5} & c_{4} & c_{3} \end{array}\right).$$

As one can observe, by increasing the Padé levels, the condition numbers grow rapidly. Therefore, the systems of equations become ill-conditioned. For computations in Table 1, $p_{0}$ was chosen in the optimal range. The very high condition number is a manifestation of the unstable and inaccurate numerical calculations. 

Table 2 shows the Toeplitz matrices for the same function, Equation (12), calculated by a different value of $p_{0}$ still in the optimal range. However, as the variation of $p_{0}$ changes the condition numbers, the condition numbers are still quite large, which is the sign of ill-conditioned systems of equations. 

For the next example, we consider f(t) that is made of two exponential decays

(14) $$f\left(t\right)=25e^{-0.05t}+e^{-0.002t}.$$

Aubard et al. [13] considered that $p_{0}=0.0043$ for this two-component function is in the optimal range. Toeplitz matrices for different Padé levels of Equation (14) are given in Table 3. Like the computation results for the three-component function, Equation (12), although $p_{0}$ was chosen in the optimal range, the condition numbers of the Toeplitz matrices are still quite large. 

Table 4 shows the Toeplitz matrices and their condition numbers for the same function at different Padé levels, where $p_{0}$ is out of the optimal range. Interestingly, the condition numbers of coefficient matrices for the $p_{0}$ value that is out of the optimal range are still large, while they are smaller than condition numbers calculated with $p_{0}$ in the optimal range.

By increasing the number of exponential modes, higher levels of Padé approximants should be calculated to reach the stable modes and, thus, by increasing the size of Toeplitz matrix, the condition numbers become very large. For example, consider the function f(t) that consists of five exponential decays

(15) $$f(t)=8560e^{-0.5t}+5650e^{-0.2t}+3725e^{-0.1t}+2358e^{-0.7t}+1350e^{-0.01t}.$$

Table 5 shows the condition numbers for different Padé levels calculated for f(t) given by Equation (15).

Consistent with the results of previous examples, increasing the Padé levels causes the rapid growth of condition numbers.

As mentioned earlier, the objective of the PL theory is to deconvolute the exponential decays from discrete experimental data f(t) measured at different time intervals. Thus, the integration by Equation (5) must be conducted numerically. To further analyze the PL theory, the entries of the coefficient matrix will be expressed in terms of the parameters of the problem. In other words, the coefficient matrix will be constructed for a general case where the function f(t) is considered in the form given by Equation (1) and expresses the entries of the coefficient matrix in terms of *α_i_* and *β_i_*. Using Equations (4) and (5), the entries of the coefficient matrix in Equation (9) are given by

(16) $$c_{k}=\frac{1}{k!}{\left(\int_{0}^{\infty }{\left(-t\right)}^{k}f(t)\exp(-pt)dt\right)}_{p=p_{0}}.$$

Now, by plugging f(t) from Equation (1), in the form of $f(t)=\overset{n}{\underset{i=1}{\sum }}\alpha_{i}\exp(\beta_{i}t)$, into Equation (16), we arrive at

(17) $$c_{k}={\left(-1\right)}^{k}\overset{n}{\underset{i=1}{\sum }}\frac{\alpha_{i}}{{\left(p_{0}-\beta_{i}\right)}^{k+1}},$$

where the conditions Re(*p*_0_) > Re(*β_i_*) for all values of *β_i_* and Re(*k*) > −1 must be satisfied. Thus, for [1/2], [2/3], and [3/4] Padé levels the coefficient matrix will be presented as

(18) $$[1/2]\to \left(\begin{array}{cc} c_{1} & c_{0}\\ c_{2} & c_{1} \end{array}\right)=\left(\begin{array}{cc} \overset{n}{\underset{i=1}{\sum }}\frac{-\alpha_{i}}{{\left(p_{0}-\beta_{i}\right)}^{2}} & \overset{n}{\underset{i=1}{\sum }}\frac{\alpha_{i}}{p_{0}-\beta_{i}}\\ \overset{n}{\underset{i=1}{\sum }}\frac{\alpha_{i}}{{\left(p_{0}-\beta_{i}\right)}^{3}} & \overset{n}{\underset{i=1}{\sum }}\frac{-\alpha_{i}}{{\left(p_{0}-\beta_{i}\right)}^{2}} \end{array}\right),$$

(19) $$\left[2/3\right]\to \left(\begin{array}{ccc} c_{2} & c_{1} & c_{0}\\ c_{3} & c_{2} & c_{1}\\ c_{4} & c_{3} & c_{2} \end{array}\right)=\left(\begin{array}{ccc} \overset{n}{\underset{i=1}{\sum }}\frac{\alpha_{i}}{{\left(p_{0}-\beta_{i}\right)}^{3}} & \overset{n}{\underset{i=1}{\sum }}\frac{-\alpha_{i}}{{\left(p_{0}-\beta_{i}\right)}^{2}} & \overset{n}{\underset{i=1}{\sum }}\frac{\alpha_{i}}{p_{0}-\beta_{i}}\\ \overset{n}{\underset{i=1}{\sum }}\frac{-\alpha_{i}}{{\left(p_{0}-\beta_{i}\right)}^{4}} & \overset{n}{\underset{i=1}{\sum }}\frac{\alpha_{i}}{{\left(p_{0}-\beta_{i}\right)}^{3}} & \overset{n}{\underset{i=1}{\sum }}\frac{-\alpha_{i}}{{\left(p_{0}-\beta_{i}\right)}^{2}}\\ \overset{n}{\underset{i=1}{\sum }}\frac{\alpha_{i}}{{\left(p_{0}-\beta_{i}\right)}^{5}} & \overset{n}{\underset{i=1}{\sum }}\frac{-\alpha_{i}}{{\left(p_{0}-\beta_{i}\right)}^{4}} & \overset{n}{\underset{i=1}{\sum }}\frac{\alpha_{i}}{{\left(p_{0}-\beta_{i}\right)}^{3}} \end{array}\right),$$

(20) $$\left[3/4\right]\to \left(\begin{array}{cccc} c_{3} & c_{2} & c_{1} & c_{0}\\ c_{4} & c_{3} & c_{2} & c_{1}\\ c_{5} & c_{4} & c_{3} & c_{2}\\ c_{6} & c_{5} & c_{4} & c_{3} \end{array}\right)=\left(\begin{array}{cccc} \overset{n}{\underset{i=1}{\sum }}\frac{-\alpha_{i}}{{\left(p_{0}-\beta_{i}\right)}^{4}} & \overset{n}{\underset{i=1}{\sum }}\frac{\alpha_{i}}{{\left(p_{0}-\beta_{i}\right)}^{3}} & \overset{n}{\underset{i=1}{\sum }}\frac{-\alpha_{i}}{{\left(p_{0}-\beta_{i}\right)}^{2}} & \overset{n}{\underset{i=1}{\sum }}\frac{\alpha_{i}}{p_{0}-\beta_{i}}\\ \overset{n}{\underset{i=1}{\sum }}\frac{\alpha_{i}}{{\left(p_{0}-\beta_{i}\right)}^{5}} & \overset{n}{\underset{i=1}{\sum }}\frac{-\alpha_{i}}{{\left(p_{0}-\beta_{i}\right)}^{4}} & \overset{n}{\underset{i=1}{\sum }}\frac{\alpha_{i}}{{\left(p_{0}-\beta_{i}\right)}^{3}} & \overset{n}{\underset{i=1}{\sum }}\frac{-\alpha_{i}}{{\left(p_{0}-\beta_{i}\right)}^{2}}\\ \overset{n}{\underset{i=1}{\sum }}\frac{-\alpha_{i}}{{\left(p_{0}-\beta_{i}\right)}^{6}} & \overset{n}{\underset{i=1}{\sum }}\frac{\alpha_{i}}{{\left(p_{0}-\beta_{i}\right)}^{5}} & \overset{n}{\underset{i=1}{\sum }}\frac{-\alpha_{i}}{{\left(p_{0}-\beta_{i}\right)}^{4}} & \overset{n}{\underset{i=1}{\sum }}\frac{\alpha_{i}}{{\left(p_{0}-\beta_{i}\right)}^{3}}\\ \overset{n}{\underset{i=1}{\sum }}\frac{\alpha_{i}}{{\left(p_{0}-\beta_{i}\right)}^{7}} & \overset{n}{\underset{i=1}{\sum }}\frac{-\alpha_{i}}{{\left(p_{0}-\beta_{i}\right)}^{6}} & \overset{n}{\underset{i=1}{\sum }}\frac{\alpha_{i}}{{\left(p_{0}-\beta_{i}\right)}^{5}} & \overset{n}{\underset{i=1}{\sum }}\frac{-\alpha_{i}}{{\left(p_{0}-\beta_{i}\right)}^{4}} \end{array}\right),$$

respectively. The determinants of the coefficient matrices for Padé levels [1/2], [2/3], and [3/4] when the number of exponential decays in f(t) are 2, 3, and 4, respectively, are given by

(21) $$Det\left(\begin{array}{cc} \overset{2}{\underset{i=1}{\sum }}\frac{-\alpha_{i}}{{\left(p_{0}-\beta_{i}\right)}^{2}} & \overset{2}{\underset{i=1}{\sum }}\frac{\alpha_{i}}{p_{0}-\beta_{i}}\\ \overset{2}{\underset{i=1}{\sum }}\frac{\alpha_{i}}{{\left(p_{0}-\beta_{i}\right)}^{3}} & \overset{2}{\underset{i=1}{\sum }}\frac{-\alpha_{i}}{{\left(p_{0}-\beta_{i}\right)}^{2}} \end{array}\right)=-\frac{\alpha_{1}\alpha_{2}{\left(\beta_{1}-\beta_{2}\right)}^{2}}{{\left(p_{0}-\beta_{1}\right)}^{3}{\left(p_{0}-\beta_{2}\right)}^{3}},$$

(22) $$\begin{array}{cc} \; & Det\left(\begin{array}{ccc} \overset{3}{\underset{i=1}{\sum }}\frac{\alpha_{i}}{{\left(p_{0}-\beta_{i}\right)}^{3}} & \overset{3}{\underset{i=1}{\sum }}\frac{-\alpha_{i}}{{\left(p_{0}-\beta_{i}\right)}^{2}} & \overset{3}{\underset{i=1}{\sum }}\frac{\alpha_{i}}{p_{0}-\beta_{i}}\\ \overset{3}{\underset{i=1}{\sum }}\frac{-\alpha_{i}}{{\left(p_{0}-\beta_{i}\right)}^{4}} & \overset{3}{\underset{i=1}{\sum }}\frac{\alpha_{i}}{{\left(p_{0}-\beta_{i}\right)}^{3}} & \overset{3}{\underset{i=1}{\sum }}\frac{-\alpha_{i}}{{\left(p_{0}-\beta_{i}\right)}^{2}}\\ \overset{3}{\underset{i=1}{\sum }}\frac{\alpha_{i}}{{\left(p_{0}-\beta_{i}\right)}^{5}} & \overset{3}{\underset{i=1}{\sum }}\frac{-\alpha_{i}}{{\left(p_{0}-\beta_{i}\right)}^{4}} & \overset{3}{\underset{i=1}{\sum }}\frac{\alpha_{i}}{{\left(p_{0}-\beta_{i}\right)}^{3}} \end{array}\right)=\\ \; & -\frac{\alpha_{1}\alpha_{2}\alpha_{3}{\left(\beta_{1}-\beta_{2}\right)}^{2}{\left(\beta_{1}-\beta_{3}\right)}^{2}{\left(\beta_{2}-\beta_{3}\right)}^{2}}{{\left(p_{0}-\beta_{1}\right)}^{5}{\left(p_{0}-\beta_{2}\right)}^{5}{\left(p_{0}-\beta_{3}\right)}^{5}}, \end{array}$$

(23) $$\begin{array}{cc} \; & Det{\left(\begin{array}{cccc} c_{3} & c_{2} & c_{1} & c_{0}\\ c_{4} & c_{3} & c_{2} & c_{1}\\ c_{5} & c_{4} & c_{3} & c_{2}\\ c_{6} & c_{5} & c_{4} & c_{3} \end{array}\right)}_{c_{k}={\left(-1\right)}^{k}\overset{4}{\underset{i=1}{\sum }}\frac{\alpha_{i}}{{\left(p_{0}-\beta_{i}\right)}^{k+1}}}=\\ \; & \frac{\alpha_{1}\alpha_{2}\alpha_{3}\alpha_{4}{\left(\beta_{1}-\beta_{2}\right)}^{2}{\left(\beta_{1}-\beta_{3}\right)}^{2}{\left(\beta_{2}-\beta_{3}\right)}^{2}{\left(\beta_{1}-\beta_{4}\right)}^{2}{\left(\beta_{2}-\beta_{4}\right)}^{2}{\left(\beta_{3}-\beta_{4}\right)}^{2}}{{\left(p_{0}-\beta_{1}\right)}^{7}{\left(p_{0}-\beta_{2}\right)}^{7}{\left(p_{0}-\beta_{3}\right)}^{7}{\left(p_{0}-\beta_{4}\right)}^{7}}. \end{array}$$

As one can observe, the coefficient matrix becomes singular if the number of exponential decays in f(t) is less than the number of the Padé level. In effect, we have

(24) $$Det\left(\begin{array}{cc} \frac{-\alpha_{1}}{{\left(p_{0}-\beta_{1}\right)}^{2}} & \frac{\alpha_{1}}{p_{0}-\beta_{1}}\\ \frac{\alpha_{1}}{{\left(p_{0}-\beta_{1}\right)}^{3}} & \frac{-\alpha_{1}}{{\left(p_{0}-\beta_{1}\right)}^{2}} \end{array}\right)=0,$$

(25) $$Det\left(\begin{array}{ccc} \overset{n}{\underset{i=1}{\sum }}\frac{\alpha_{i}}{{\left(p_{0}-\beta_{i}\right)}^{3}} & \overset{n}{\underset{i=1}{\sum }}\frac{-\alpha_{i}}{{\left(p_{0}-\beta_{i}\right)}^{2}} & \overset{n}{\underset{i=1}{\sum }}\frac{\alpha_{i}}{p_{0}-\beta_{i}}\\ \overset{n}{\underset{i=1}{\sum }}\frac{-\alpha_{i}}{{\left(p_{0}-\beta_{i}\right)}^{4}} & \overset{n}{\underset{i=1}{\sum }}\frac{\alpha_{i}}{{\left(p_{0}-\beta_{i}\right)}^{3}} & \overset{n}{\underset{i=1}{\sum }}\frac{-\alpha_{i}}{{\left(p_{0}-\beta_{i}\right)}^{2}}\\ \overset{n}{\underset{i=1}{\sum }}\frac{\alpha_{i}}{{\left(p_{0}-\beta_{i}\right)}^{5}} & \overset{n}{\underset{i=1}{\sum }}\frac{-\alpha_{i}}{{\left(p_{0}-\beta_{i}\right)}^{4}} & \overset{n}{\underset{i=1}{\sum }}\frac{\alpha_{i}}{{\left(p_{0}-\beta_{i}\right)}^{3}} \end{array}\right)=0,\;for\;n<3,$$

(26) $$Det\left(\begin{array}{cccc} \overset{n}{\underset{i=1}{\sum }}\frac{-\alpha_{i}}{{\left(p_{0}-\beta_{i}\right)}^{4}} & \overset{n}{\underset{i=1}{\sum }}\frac{\alpha_{i}}{{\left(p_{0}-\beta_{i}\right)}^{3}} & \overset{n}{\underset{i=1}{\sum }}\frac{-\alpha_{i}}{{\left(p_{0}-\beta_{i}\right)}^{2}} & \overset{n}{\underset{i=1}{\sum }}\frac{\alpha_{i}}{p_{0}-\beta_{i}}\\ \overset{n}{\underset{i=1}{\sum }}\frac{\alpha_{i}}{{\left(p_{0}-\beta_{i}\right)}^{5}} & \overset{n}{\underset{i=1}{\sum }}\frac{-\alpha_{i}}{{\left(p_{0}-\beta_{i}\right)}^{4}} & \overset{n}{\underset{i=1}{\sum }}\frac{\alpha_{i}}{{\left(p_{0}-\beta_{i}\right)}^{3}} & \overset{n}{\underset{i=1}{\sum }}\frac{-\alpha_{i}}{{\left(p_{0}-\beta_{i}\right)}^{2}}\\ \overset{n}{\underset{i=1}{\sum }}\frac{-\alpha_{i}}{{\left(p_{0}-\beta_{i}\right)}^{6}} & \overset{n}{\underset{i=1}{\sum }}\frac{\alpha_{i}}{{\left(p_{0}-\beta_{i}\right)}^{5}} & \overset{n}{\underset{i=1}{\sum }}\frac{-\alpha_{i}}{{\left(p_{0}-\beta_{i}\right)}^{4}} & \overset{n}{\underset{i=1}{\sum }}\frac{\alpha_{i}}{{\left(p_{0}-\beta_{i}\right)}^{3}}\\ \overset{n}{\underset{i=1}{\sum }}\frac{\alpha_{i}}{{\left(p_{0}-\beta_{i}\right)}^{7}} & \overset{n}{\underset{i=1}{\sum }}\frac{-\alpha_{i}}{{\left(p_{0}-\beta_{i}\right)}^{6}} & \overset{n}{\underset{i=1}{\sum }}\frac{\alpha_{i}}{{\left(p_{0}-\beta_{i}\right)}^{5}} & \overset{n}{\underset{i=1}{\sum }}\frac{-\alpha_{i}}{{\left(p_{0}-\beta_{i}\right)}^{4}} \end{array}\right)=0,\;for\;n<4.$$

This analysis indicates that achieving the higher levels of Padé levels can result in singular Toeplitz matrices. Moreover, the calculations show that the coefficient matrices become rank-deficient, and, in fact, the rank of the coefficient matrix equals the number of exponential decays in function f(t). For example, we have

(27) $$Rank\left(\begin{array}{cc} \overset{n}{\underset{i=1}{\sum }}\frac{-\alpha_{i}}{{\left(p_{0}-\beta_{i}\right)}^{2}} & \overset{n}{\underset{i=1}{\sum }}\frac{\alpha_{i}}{p_{0}-\beta_{i}}\\ \overset{n}{\underset{i=1}{\sum }}\frac{\alpha_{i}}{{\left(p_{0}-\beta_{i}\right)}^{3}} & \overset{n}{\underset{i=1}{\sum }}\frac{-\alpha_{i}}{{\left(p_{0}-\beta_{i}\right)}^{2}} \end{array}\right)=n,\;for\;n\leq 2,$$

(28) $$Rank\left(\begin{array}{ccc} \overset{n}{\underset{i=1}{\sum }}\frac{\alpha_{i}}{{\left(p_{0}-\beta_{i}\right)}^{3}} & \overset{n}{\underset{i=1}{\sum }}\frac{-\alpha_{i}}{{\left(p_{0}-\beta_{i}\right)}^{2}} & \overset{n}{\underset{i=1}{\sum }}\frac{\alpha_{i}}{p_{0}-\beta_{i}}\\ \overset{n}{\underset{i=1}{\sum }}\frac{-\alpha_{i}}{{\left(p_{0}-\beta_{i}\right)}^{4}} & \overset{n}{\underset{i=1}{\sum }}\frac{\alpha_{i}}{{\left(p_{0}-\beta_{i}\right)}^{3}} & \overset{n}{\underset{i=1}{\sum }}\frac{-\alpha_{i}}{{\left(p_{0}-\beta_{i}\right)}^{2}}\\ \overset{n}{\underset{i=1}{\sum }}\frac{\alpha_{i}}{{\left(p_{0}-\beta_{i}\right)}^{5}} & \overset{n}{\underset{i=1}{\sum }}\frac{-\alpha_{i}}{{\left(p_{0}-\beta_{i}\right)}^{4}} & \overset{n}{\underset{i=1}{\sum }}\frac{\alpha_{i}}{{\left(p_{0}-\beta_{i}\right)}^{3}} \end{array}\right)=0,\;for\;n\leq 3,$$

(29) $$Rank{\left(\begin{array}{cccc} c_{3} & c_{2} & c_{1} & c_{0}\\ c_{4} & c_{3} & c_{2} & c_{1}\\ c_{5} & c_{4} & c_{3} & c_{2}\\ c_{6} & c_{5} & c_{4} & c_{3} \end{array}\right)}_{c_{k}={\left(-1\right)}^{k}\overset{n}{\underset{i=1}{\sum }}\frac{\alpha_{i}}{{\left(p_{0}-\beta_{i}\right)}^{k+1}},}=n,\;for\;n\leq 4.$$

It means that the coefficient matrix for each Padé level higher than the number of exponential decays is rank-deficient, which is consistent with the determinants of the coefficient matrices. 

The results presented herein indicate that PL is an ill-posed method for the separation of exponential functions. Therefore, in contrast to the previous thought that the method is taking advantage of the properties of analyticity of Laplace transform to deal with the ill-posed problem of separation of exponentials, [9] the computation of Padé approximants generates ill-conditioned systems of equations. Although the PL method proposes a powerful numerical procedure for deconvolution of the exponential modes, it is likely to encounter ill-conditioned problems attributable to the ill-posedness of Padé table computations. In the next section, we will exploit a numerical algorithm to regularize the PL method and demonstrate that this algorithm can successfully resolve the ill-posedness of the PL numerical procedure.

## 4. Regularization of the PL Method

To resolve the ill-posedness of the PL numerical procedure, one must regularize this method. As mentioned in Section 2, after considering the condition $b_{0}=1$, Equation (6) will be expressed as an ill-conditioned system of linear equations. It is important to note that the Longman algorithm [5] also implements the same condition $b_{0}=1$ (see Equation (7) in [5]). Therefore, the instability reported by Tang and Norris [6] for using the Longman algorithm might be attributed to using the same coefficient conditions.

Knowing the origin of ill-posedness, one may regularize the PL method by changing the coefficient condition $b_{0}=1$, and reconstruct the system of equations in a way that eliminates the ill-conditioning.

Recently, Gonnet et al. [16] proposed a numerical algorithm for computation of the Padé table using singular value decomposition (SVD). In this numerical algorithm, instead of using the coefficient condition $b_{0}=1$, they considered

(30) $${\left\|\left(\begin{array}{c} b_{0}\\ b_{1}\\ \vdots \\ b_{n} \end{array}\right)\right\|}_{2}=1,$$

where ${\left(\begin{array}{cccc} b_{0} & b_{1} & \cdots & b_{n} \end{array}\right)}^{T}$ is a vector whose components are the coefficients of the polynomial in the denominator of the Padé approximant and ${\left\|\cdot \right\|}_{2}$ is the vector 2-norm operator. After computations, the output of the numerical algorithm presents a polynomial in the denominator of the Padé approximants in the form of

(31) $$Q(x)=1+b_{1}x+b_{2}x^{2}+\cdots +b_{n}x^{n}.$$

Using the normalization condition, Equation (30), in computation of the Padé table helps to eliminate the ill-conditioning problem. In effect, the numerical algorithm proposed by Gonnet et al. [16] can regularize the ill-posed problem of Padé computations. Hereafter, we call the regularized PL method RPL, which represents the PL method where Padé table is computed using the SVD solver developed by Gonnet et al. [16]. In the following, we show the capability of RPL in separation of exponentials. Table 6 shows the results of the deconvolution of generated data points of the function f(t) in Equation (12) using RPL. As shown in Table 6, three stable modes appear in [4/5] and [5/6] Padé approximants. It is expected that these stable modes remain in the computations after increasing the Padé levels. On the other hand, considering the rapid growth of condition number of Toeplitz matrices, as shown earlier, achieving high levels of Padé approximants is not possible without regularization. 

Table 7 shows the results of deconvolution of generated data points of the function f(t) in Equation (12) using RPL in [10/11] and [11/12] Padé approximants. 

The stable modes are shown in bold. It should be noted that by increasing the Padé levels, round-off errors might affect the accuracy.

Table 8 and Table 9 show the deconvolution results for generated data points of the function f(t) in Equation (12) using RPL when $p_{0}$ values are out of the optimal range. The stable modes are shown in bold.

When $p_{0}$ is out of the optimal range, the RPL can separate exponential decays; however, this can only be done at the expense of calculating higher levels of Padé approximants. The computation results presented in Table 8 and Table 9 demonstrate that when $p_{0}$ is out of the optimal range, stable modes appear at higher levels of Padé approximants.

Table 10 and Table 11 show the results of deconvolution of the function f(t) in Equations (14) and (15), respectively. In both examples $p_{0}$ was considered out of the optimal range. The computation results exhibit the capability of RPL in deconvolution of these functions.

### Deconvolution of Noisy Data by RPL

**Figure 1:**
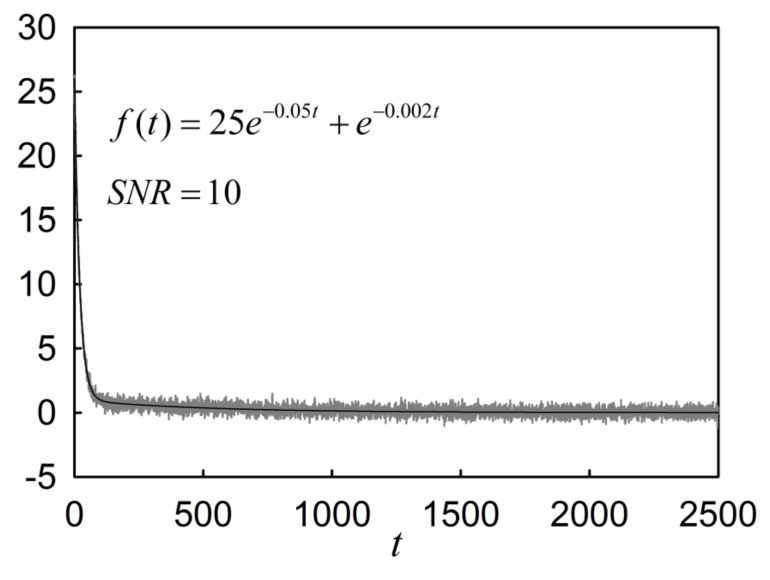
The two-component function f(t) before and after adding a white Gaussian noise with SNR = 10.

To show the capability of regularization in tackling the deconvolution process, consider a two-component function, f(t) in Equation (14), and analyze it with RPL after adding white Gaussian noise with the signal-to-noise ratio of SNR = 10 dB. Figure 1 shows the plots of function f(t) before and after adding the white Gaussian noise.

Table 12 shows the result of deconvolution of the noisy data shown in Figure 1 by RPL. The deconvolution results indicate that the RPL can find the exponential decays from the data points perturbed by a noise with SNR = 10. However, by increasing the Padé levels, the results start to deviate from the actual values. This deviation is attributable to the cumulative errors involved in the numerical integration of the noisy data. 

As explained in Section 2, to calculate the Taylor expansion coefficients, numerical integration must be performed on the discrete data points. The noise results in cumulative error in numerical integration. For low levels of Padé approximants, where a small number of coefficients need to be calculated, the RPL is capable of deconvolution of the data points; however, by increasing the number of levels, the numerical error results in deviation from the actual values.

**Figure 2:**
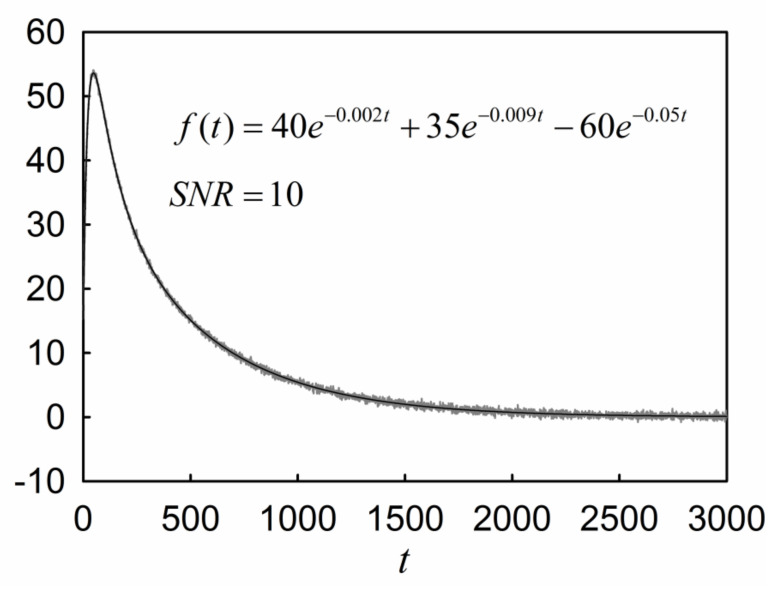
The three-component function f(t) before and after adding white Gaussian noise with SNR = 10.

Figure 2 shows the plots of function f(t)

(32) $$f\left(t\right)=40e^{-0.002t}+35e^{-0.009t}-60e^{-0.05t},$$

before and after adding white Gaussian noise of SNR = 10 dB.

Table 13 and Table 14 give the deconvolution results for Equation (32) before and after adding the noise, respectively. Like the results we found in the case of Equation (14), the RPL is capable of finding the exponential modes after disturbing the data points. The deviation observed in the results shown in Table 14 is attributable to perturbation of data with the noise.

## 5. Conclusions

The Padé–Laplace (PL) method is a powerful numerical scheme for deconvolution of Maxwellian modes from stress relaxation data of polymers obtained in discrete time intervals. The PL method needs only one parameter to perform the computations and does not require any initial guesses for the number of modes and parameters (amplitude and exponents) associated with each mode. The amplitudes and their corresponding exponents convey important information relating to the rheological behavior of polymers. In effect, the relaxation spectrum is the fingerprint of any polymer that is necessary to formulate a constitutive equation and can be used to predict its rheological behavior. A crucial step in this numerical procedure is constructing the Padé approximants that is an ill-posed problem. Since 1987, when the PL method was developed for separation of exponentials, the potential problem of ill-posedness attributable to the computation of the Padé table has been overlooked. In this paper, it was shown that the computation of the Padé approximants can result in ill-conditioned systems of equations. Therefore, it was shown that, apart from its elegant mathematical structure, the PL method that was believed to be able to solve the ill-posed problem of separation of exponentials using the properties of Laplace transform of an analytic function [9] can produce ill-conditioned systems of equations. As numerical computations demonstrate, the condition number of a matrix whose entries are the coefficient of Taylor expansion grows rapidly. A regularization of this method is possible by reconstructing the system of equations and using singular value decomposition (SVD) for computation of the Padé table. After regularization, the numerical computation indicates that the PL method can deconvolute data points even when $p_{0}$, the only input parameter of the method, is chosen out of its optimal range. However, this occurs at the expense of calculating more levels of Padé approximants to achieve the stable modes. The analysis shown in this paper recommends applying the same regularization method in cases where the extended version of the PL method [9] was used to deconvolute experimental data. Although the focus of this paper in terms of application was on the deconvolution of viscoelastic spectrum of polymers, the results of this work will be fruitful in other areas such as analysis of chemical relaxation signals [13], voltage decays [14], fluorescence intensity decay [17,18], NMR relaxation data [19,20,21], and transient electric birefringence decay [22].

## Figures and Tables

**Table 1:** Toeplitz matrices associated with different Padé levels and their condition numbers for function $f(t)=150e^{-0.004t}+19e^{-0.04t}+30e^{-0.06t}$ when $p_{0}=0.04$.


Padé approximant [0/1]

3947.51750294869

2-norm condition number = 1
Infinity-norm condition number = 1


Padé approximant [1/2]

$\left(\begin{array}{cc} -83431.5084764036 & 3947.51750294869\\ 1828003.39329228 & -83431.5084764036 \end{array}\right)$

2-norm condition number = 1.31 × 10^4^
Infinity-norm condition number = 1.43 × 10^4^


Padé approximant [2/3]

$\left(\begin{array}{ccc} 1828003.39329228 & -83431.5084764036 & 3947.51750294869\\ -40784187.1136812 & 1828003.39329228 & -83431.5084764036\\ 918351059.166385 & -40784187.1136812 & 1828003.39329228 \end{array}\right)$

2-norm condition number = 1.33 × 10^8^
Infinity-norm condition number = 1.55 × 10^4^

Padé approximant [3/4]
$\left(\begin{array}{cccc} -40784187.1136812 & 1828003.39329228 & -83431.5084764036 & 3947.51750294869\\ 918351059.166385 & -40784187.1136812 & 1828003.39329228 & -83431.5084764036\\ -20774131959.9484 & 918351059.166385 & -40784187.1136812 & 1828003.39329228\\ 471016279508.57 & -20774131959.9484 & 918351059.166385 & -40784187.1136812 \end{array}\right)$

2-norm condition number = 6.85 × 10^10^
Infinity-norm condition number = 8.05 × 10^10^


**Table 2:** Toeplitz matrices associated with different Padé levels and their condition numbers for function $f(t)=150e^{-0.004t}+19e^{-0.04t}+30e^{-0.06t}$ when $p_{0}=0.02$.


Padé approximant [0/1]

6942.26163687135

2-norm condition number = 1
Infinity-norm condition number = 1


Padé approximant [1/2]

$\left(\begin{array}{cc} -270365.362556016 & 6942.26163687135\\ 10997251.1276327 & -270365.362556016 \end{array}\right)$

2-norm condition number = 3.73 × 10^4^
Infinity-norm condition number = 3.91 × 10^4^


Padé approximant [2/3]

$\left(\begin{array}{ccc} 10997251.1276327 & -270365.362556016 & 6942.26163687135\\ -454310740.051794 & 10997251.1276327 & -270365.362556016\\ 18871600618.0707 & -454310740.051794 & 10997251.1276327 \end{array}\right)$

2-norm condition number = 1.96 × 10^9^
Infinity-norm condition number = 2.18 × 10^9^

Padé approximant [3/4]

$\left(\begin{array}{cccc} -454310740.051794 & 10997251.1276327 & -270365.362556016 & 6942.26163687135\\ 18871600618.0707 & -454310740.051794 & 10997251.1276327 & -270365.362556016\\ -785438809700.987 & 18871600618.0707 & -454310740.051794 & 10997251.1276327\\ 32713098312245.6 & -785438809700.987 & 18871600618.0707 & -454310740.051794 \end{array}\right)$

2-norm condition number = 5.77 × 10^12^
Infinity-norm condition number = 6.69 × 10^12^


**Table 3:** Toeplitz matrices associated with different Padé levels and their condition numbers for function $f(t)=25e^{-0.05t}+e^{-0.002t}$ when $p_{0}=0.0043$.


Padé approximant [0/1]

619.163704402595

2-norm condition number = 1
Infinity-norm condition number = 1


Padé approximant [1/2]

$\left(\begin{array}{cc} -33673.5768069156 & 619.163704402595\\ 4155316.031534 & -33673.5768069156 \end{array}\right)$

2-norm condition number = 1.2 × 10^4^
Infinity-norm condition number = 1.22 × 10^4^


Padé approximant [2/3]

$\left(\begin{array}{ccc} 4155316.031534 & -33673.5768069156 & 619.163704402595\\ -637604141.531504 & 4155316.031534 & -33673.5768069156\\ 100766089551.323 & -637604141.531504 & 4155316.031534 \end{array}\right)$

2-norm condition number = 3.38 × 10^10^
Infinity-norm condition number = 3.59 × 10^10^

Padé approximant [3/4]

$\left(\begin{array}{cccc} -637604141.531504 & 4155316.031534 & -33673.5768069156 & 619.163704402595\\ 100766089551.323 & -637604141.531504 & 4155316.031534 & -33673.5768069156\\ -15968480782012.7 & 100766089551.323 & -637604141.531504 & 4155316.031534\\ 2.52675015701706e+15 & -15968480782012.7 & 100766089551.323 & -637604141.531504 \end{array}\right)$

2-norm condition number = 3.42 × 10^15^
Infinity-norm condition number = 3.65 × 10^15^


**Table 4:** Toeplitz matrices associated with different Padé levels and their condition numbers for function $f(t)=25e^{-0.05t}+e^{-0.002t}$ when $p_{0}=0.1$.


Padé approximant [0/1]

176.550830813744

2-norm condition number = 1
Infinity-norm condition number = 1


Padé approximant [1/2]

$\left(\begin{array}{cc} -1206.68647171976 & 176.550830813744\\ 8349.72873932373 & -1206.68647171976 \end{array}\right)$

2-norm condition number = 4.02 × 10^3^
Infinity-norm condition number = 5.06 × 10^3^


Padé approximant [2/3]

$\left(\begin{array}{ccc} 8349.72873932373 & -1206.68647171976 & 176.550830813744\\ -58621.1725640864 & 8349.72873932373 & -1206.68647171976\\ 419791.187990069 & -58621.1725640864 & 8349.72873932373 \end{array}\right)$

2-norm condition number = 1.29 × 10^6^
Infinity-norm condition number = 1.75 × 10^6^

Padé approximant [3/4]

$\left(\begin{array}{cccc} -58621.1725640864 & 8349.72873932373 & -1206.68647171976 & 176.550830813744\\ 419791.187990069 & -58621.1725640864 & 8349.72873932373 & -1206.68647171976\\ -3082758.76213702 & 419791.187990069 & -58621.1725640864 & 8349.72873932373\\ 23337517.6524446 & -3082758.76213702 & 419791.187990069 & -58621.1725640864 \end{array}\right)$

2-norm condition number = 4.94 × 10^7^
Infinity-norm condition number = 6.31 × 10^7^


**Table 5:** Condition numbers of Toeplitz matrices associated with different Padé levels for function $f(t)=8560e^{-0.5t}+5650e^{-0.2t}+3725e^{-0.1t}+2358e^{-0.7t}+1350e^{-0.01t}$ when $p_{0}=1$.

Padé Approximant	2-Norm Condition Number	Infinity-Norm Condition Number
[0/1]	1	1
[1/2]	146	177
[2/3]	2.97 × 10^3^	4.53 × 10^3^
[3/4]	1.14 × 10^5^	1.87 × 10^5^
[4/5]	6.31 × 10^5^	1.09 × 10^6^
[5/6]	4.28 × 10^7^	7.73 × 10^7^
[6/7]	8.14 × 10^9^	1.53 × 10^10^
[7/8]	1.15 × 10^13^	2.3 × 10^13^
[8/9]	3.96 × 10^15^	8.12 × 10^15^
[9/10]	1.56 × 10^17^	2.13 × 10^17^

**Table 6:** Deconvolution of three-component function $f(t)=150e^{-0.004t}+19e^{-0.04t}+30e^{-0.06t}$ using RPL when $p_{0}=0.04$.

Level	Re(beta)	Im(beta)	Re(alpha)	Im(alpha)
[0/1]	−7.3144688 × 10^−^^3^	0.0000000 × 10^0^	1.8677469 × 10^2^	0.0000000 × 10^0^
[1/2] [1/2]	−5.4778310 × 10^−^^2^ −4.0862030 × 10^−^^3^	0.0000000 × 10^0^ 0.0000000 × 10^0^	4.7728408 × 10^1^ 1.5183015 × 10^2^	0.0000000 × 10^0^ 0.0000000 × 10^0^
[2/3] [2/3] [2/3]	−3.1173129 × 10^0^ −5.0003912 × 10^−^^2^ −4.0097848 × 10^−^^3^	0.0000000 × 10^0^ 0.0000000 × 10^0^ 0.0000000 × 10^0^	2.4292682 × 10^1^ 4.7223963 × 10^1^ 1.5029938 × 10^2^	0.0000000 × 10^0^ 0.0000000 × 10^0^ 0.0000000 × 10^0^
[3/4] [3/4] [3/4] [3/4]	5.3238003 × 10^−^^1^ −5.4445912 × 10^−^^2^ −3.3152082 × 10^−^^2^ −3.9990211 × 10^−^^3^	0.0000000 × 10^0^ 0.0000000 × 10^0^ 0.0000000 × 10^0^ 0.0000000 × 10^0^	−1.7648630 × 10^0^ 4.1242607 × 10^1^ 7.2435270 × 10^0^ 1.4995893 × 10^2^	0.0000000 × 10^0^ 0.0000000 × 10^0^ 0.0000000 × 10^0^ 0.0000000 × 10^0^
[4/5] [4/5] [4/5] [4/5] [4/5]	−1.6210147 × 10^−^^2^ −1.6210147 × 10^−^^2^ **−6.0000000 × 10^−^^2^** **−4.0000000 × 10^−^^2^** **−4.0000000 × 10^−^^3^**	7.7526429 × 10^0^ −7.7526429 × 10^0^ 0.0000000 × 10^0^ 0.0000000 × 10^0^ 0.0000000 × 10^0^	4.9834776 × 10^2^ 4.9834776 × 10^2^ **3.0000001 × 10^1^** **1.8999999 × 10^1^** **1.5000000 × 10^2^**	2.1295890 × 10^−^^2^ −2.1295890 × 10^−^^2^ 0.0000000 × 10^0^ 0.0000000 × 10^0^ 0.0000000 × 10^0^
[5/6] [5/6] [5/6] [5/6] [5/6] [5/6]	−1.9041159 × 10^−^^2^ −1.9041159 × 10^−^^2^ −6.7767944 × 10^−^^1^ **−5.9999995 × 10^−^^1^** **−3.9999998 × 10^−^^2^** **−4.0000000 × 10^−^^3^**	7.7689403 × 10^0^ −7.7689403 × 10^0^ 0.0000000 × 10^0^ 0.0000000 × 10^0^ 0.0000000 × 10^0^ 0.0000000 × 10^0^	5.0048358 × 10^2^ 5.0048358 × 10^2^ 5.9734657 × 10^−^^4^ **3.0000007 × 10^1^** **1.8999993 × 10^1^** **1.5000000 × 10^2^**	2.0717494 × 10^−^^1^ −2.0717494 × 10^−^^1^ 0.0000000 × 10^0^ 0.0000000 × 10^0^ 0.0000000 × 10^0^ 0.0000000 × 10^0^

**Table 7:** High levels of Padé approximants in deconvolution of three-component function $f(t)=150e^{-0.004t}+19e^{-0.04t}+30e^{-0.06t}$ using RPL when $p_{0}=0.04$.

Level	Re(beta)	Im(beta)	Re(alpha)	Im(alpha)
[10/11] [10/11] [10/11] [10/11] [10/11] [10/11] [10/11] [10/11] [10/11] [10/11] [10/11]	−2.1324889 × 10^−^^2^ −2.1324889 × 10^−^^2^ 1.8612450 × 10^0^ 1.0175524 × 10^−^^1^ **−5.9999997 × 10^−2^** **−3.9999998 × 10^−2^** 4.6051089 × 10^−2^ 4.6051089 × 10^−2^ **−4.0000000 × 10^−3^** 3.2810383 × 10^−3^ 3.2810383 × 10^−3^	7.7191644 × 10^0^ −7.7191644 × 10^0^ 0.0000000 × 10^0^ 0.0000000 × 10^0^ 0.0000000 × 10^0^ 0.0000000 × 10^0^ 2.7461804 × 10^−^^2^ −2.7461804 × 10^−^^2^ 0.0000000 × 10^0^ 1.3004577 × 10^−^^2^ −1.3004577 × 10^−^^2^	4.9388824 × 10^2^ 4.9388824 × 10^2^ −1.9332353 × 10^−^^2^ −1.5513358 × 10^−^^10^ **3.0000004** **× 10^1^** **1.8999995** **× 10^1^** −2.8790985 × 10^−^^10^ −2.8790985 × 10^−^^10^ **1.5000000** **× 10^2^** 3.4151907 × 10^−9^ 3.4151907 × 10^−9^	3.8817019 × 10^−^^1^ −3.8817019 × 10^−^^1^ 0.0000000 × 10^0^ 0.0000000 × 10^0^ 0.0000000 × 10^0^ 0.0000000 × 10^0^ 4.1379327 × 10^−^^10^ −4.1379327 × 10^−10^ 0.0000000 × 10^0^ 5.8441591 × 10^−9^ −5.8441591 × 10^−9^
[11/12] [11/12] [11/12] [11/12] [11/12] [11/12] [11/12] [11/12] [11/12] [11/12] [11/12] [11/12]	−4.7391958 × 10^−^^2^ −4.7391958 × 10^−^^2^ 4.5673491 × 10^0^ 1.1502705 × 10^−^^1^ **−5.9999997 × 10^−^^2^** **−3.9999998 × 10^−^^2^** 4.2291942 × 10^−^^2^ 4.2291942 × 10^−^^2^ 3.3846007 × 10^−^^3^ 3.3846007 × 10^−^^3^ **−4.0000000 × 10^−^^3^** 4.4403675 × 10^−^^2^	7.6874054 × 10^0^ −7.6874054 × 10^0^ 0.0000000 × 10^0^ 0.0000000 × 10^0^ 0.0000000 × 10^0^ 0.0000000 × 10^0^ 2.6291712 × 10^−^^2^ −2.6291712 × 10^−^^2^ 1.2973154 × 10^−^^2^ −1.2973154 × 10^−^^2^ 0.0000000 × 10^0^ 0.0000000 × 10^0^	4.8822528 × 10^2^ 4.8822528 × 10^2^ −1.2573896 × 10^0^ −1.2626689 × 10^−10^ **3.0000004** **× 10^1^** **1.8999995** **× 10^1^** −3.1523708 × 10^−10^ −3.1523708 × 10^−10^ 3.3215604 × 10^−9^ 3.3215604 × 10^−9^ **1.5000000** **× 10^2^** −6.9425412 × 10^−10^	3.0558821 × 10^0^ −3.0558821 × 10^0^ 0.0000000 × 10^0^ 0.0000000 × 10^0^ 0.0000000 × 10^0^ 0.0000000 × 10^0^ 4.8228821 × 10^−10^ −4.8228821 × 10^−10^ 5.8739554 × 10^−9^ −5.8739554 × 10^−9^ 0.0000000 × 10^0^ 0.0000000 × 10^0^

**Table 8:** Deconvolution of three-component function $f(t)=150e^{-0.004t}+19e^{-0.04t}+30e^{-0.06t}$ using RPL when $p_{0}=2$.

Level	Re(beta)	Im(beta)	Re(alpha)	Im(alpha)
[10/11] [10/11] [10/11] [10/11] [10/11] [10/11] [10/11] [10/11] [10/11] [10/11] [10/11]	−2.7077041 × 10^1^ −2.7077041 × 10^1^ −6.1805903 × 10^0^ −6.1805903 × 10^0^ −2.5554003 × 10^−1^ −2.5554003 × 10^−1^ −1.5365995 × 10^−2^ −1.5365995 × 10^−2^ **−6.0005910** **× 10^−2^** **−4.0019812** **× 10^−2^** **−4.0002474** **× 10^−3^**	9.1626768 × 10^1^ −9.1626768 × 10^1^ 2.1050940 × 10^1^ −2.1050940 × 10^1^ 1.2306962 × 10^1^ −1.2306962 × 10^1^ 6.2841161 × 10^0^ −6.2841161 × 10^0^ 0.0000000 × 10^0^ 0.0000000 × 10^0^ 0.0000000 × 10^0^	7.2543691 × 10^3^ 7.2543691 × 10^3^ 5.1467900 × 10^2^ 5.1467900 × 10^2^ 1.8078545 × 10^2^ 1.8078545 × 10^2^ 1.9913166 × 10^2^ 1.9913166 × 10^2^ **2.9976264** **× 10^1^** **1.9019637** **× 10^1^** **1.5000415** **× 10^2^**	7.8190760 × 10^2^ −7.8190760 × 10^2^ 3.8373116 × 10^2^ −3.8373116 × 10^2^ 4.6513878 × 10^1^ −4.6513878 × 10^1^ −1.9550354 × 10^−1^ 1.9550354 × 10^−1^ 0.0000000 × 10^0^ 0.0000000 × 10^0^ 0.0000000 × 10^0^
[11/12] [11/12] [11/12] [11/12] [11/12] [11/12] [11/12] [11/12] [11/12] [11/12] [11/12] [11/12]	1.9960781 × 10^2^ −1.0407991 × 10^1^ −1.0407991 × 10^1^ −3.5638166 × 10^−1^ −3.5638166 × 10^−1^ −1.5318715 × 10^−2^ −1.5318715 × 10^−2^ −5.7955252 × 10^−1^ −5.7955252 × 10^−1^ **−5.9916572** **× 10^−2^** **−3.9744843** **× 10^−2^** **−3.9973272** **× 10^−3^**	0.0000000 × 10^0^ 2.3945985 × 10^1^ −2.3945985 × 10^1^ 1.2371355 × 10^1^ −1.2371355 × 10^1^ 6.2841195 × 10^0^ −6.2841195 × 10^0^ 1.1399858 × 10^0^ −1.1399858 × 10^0^ 0.0000000 × 10^0^ 0.0000000 × 10^0^ 0.0000000 × 10^0^	−1.0851438 × 10^4^ 7.6825966 × 10^2^ 7.6825966 × 10^2^ 1.9394604 × 10^2^ 1.9394604 × 10^2^ 1.9912256 × 10^2^ 1.9912256 × 10^2^ −4.0087042 × 10^−7^ −4.0087042 × 10^−7^ **3.0311253** **× 10^1^** **1.8736139** **× 10^1^** **1.4994814** **× 10^2^**	0.0000000 × 10^0^ 9.8681718 × 10^2^ −9.8681718 × 10^2^ 5.7945460 × 10^1^ −5.7945460 × 10^1^ −2.152848 × 10^−1^ 2.1528487 × 10^−1^ 5.6765099 × 10^−7^ −5.6765099 × 10^−7^ 0.0000000 × 10^0^ 0.0000000 × 10^0^ 0.0000000 × 10^0^
[12/13] [12/13] [12/13] [12/13] [12/13] [12/13] [12/13] [12/13] [12/13] [12/13] [12/13] [12/13] [12/13]	1.7618498 × 10^2^ −9.9833268 × 10^0^ −9.9833268 × 10^0^ −3.4500329 × 10^−1^ −3.4500329 × 10^−1^ −1.5318064 × 10^−2^ −1.5318064 × 10^−2^ 1.1196162 × 10^0^ 1.1196162 × 10^0^ −2.6054392 × 10^−1^ **−6.000049** **× 10^−2^** **−3.9997497** **× 10^−2^** **−3.9999174** **× 10^−3^**	0.0000000 × 10^0^ 2.4442457 × 10^1^ −2.4442457 × 10^1^ 1.2393825 × 10^1^ −1.2393825 × 10^1^ 6.2840948 × 10^0^ −6.2840948 × 10^0^ 2.1693108 × 10^0^ −2.1693108 × 10^0^ 0.0000000 × 10^0^ 0.0000000 × 10^0^ 0.0000000 × 10^0^ 0.0000000 × 10^0^	−8.9677724 × 10^3^ 8.3504040 × 10^2^ 8.3504040 × 10^2^ 1.9620433 × 10^2^ 1.9620433 × 10^2^ 1.9911357 × 10^2^ 1.9911357 × 10^2^ −1.0252367 × 10^−10^ −1.0252367 × 10^−10^ −8.7665167 × 10^−6^ **3.0003785** **× 10^1^** **1.9006856** **× 10^1^** **1.5005297** **× 10^2^**	0.0000000 × 10^0^ 9.5099219 × 10^2^ −9.5099219 × 10^2^ 5.5609160 × 10^1^ −5.5609160 × 10^1^ −2.1027043 × 10^−1^ 2.1027043 × 10^−1^ 5.1378617 × 10^−11^ −5.1378617 × 10^−11^ 0.0000000 × 10^0^ 0.0000000 × 10^0^ 0.0000000 × 10^0^ 0.0000000 × 10^0^

**Table 9:** Deconvolution of three-component function $f(t)=150e^{-0.004t}+19e^{-0.04t}+30e^{-0.06t}$ using RPL when $p_{0}=3$.

Level	Re(beta)	Im(beta)	Re(alpha)	Im(alpha)
[16/17] [16/17] [16/17] [16/17] [16/17] [16/17] [16/17] [16/17] [16/17] [16/17] [16/17] [16/17] [16/17] [16/17] [16/17] [16/17] [16/17]	−5.5030888 × 10^1^ −5.5030888 × 10^1^ −2.9897761 × 10^0^ −2.9897761 × 10^0^ −3.4405403 × 10^−2^ −3.4405403 × 10^−2^ 8.8670363 × 10^0^ −1.4329960 × 10^−1^ −1.4329960 × 10^−1^ −1.2296947 × 10^−2^ −1.2296947 × 10^−2^ **−6.0183356 × 10^−2^** **−4.0560106 × 10^−2^** **−4.0063800 × 10^−3^** 3.6260369 × 10^0^ 2.9143057 × 10^0^ 3.0000759 × 10^0^	8.2151753 × 10^1^ −8.2151753 × 10^1^ 2.0046116 × 10^1^ −2.0046116 × 10^1^ 1.2522466 × 10^1^ −1.2522466 × 10^1^ 0.0000000 × 10^0^ 6.2347883 × 10^0^ −6.2347883 × 10^0^ 6.2827305 × 10^0^ −6.2827305 × 10^0^ 0.0000000 × 10^0^ 0.0000000 × 10^0^ 0.0000000 × 10^0^ 0.0000000 × 10^0^ 0.0000000 × 10^0^ 0.0000000 × 10^0^	1.0428004 × 10^4^ 1.0428004 × 10^4^ 3.8418716 × 10^2^ 3.8418716 × 10^2^ 1.9188035 × 10^2^ 1.9188035 × 10^2^ −2.2666940 × 10^−8^ 4.4928375 × 10^0^ 4.4928375 × 10^0^ 1.9448884 × 10^2^ 1.9448884 × 10^2^ **2.9308374** **× 10^1^** **1.9596862** **× 10^1^** **1.5009758** **× 10^2^** −5.5123535 × 10^−14^ −1.1941466 × 10^−13^ −1.0784734 × 10^−13^	5.7438302 × 10^3^ −5.7438302 × 10^3^ 2.5860369 × 10^2^ −2.5860369 × 10^2^ 7.4973331 × 10^0^ −7.4973331 × 10^0^ 0.0000000 × 10^0^ −2.1898894 × 10^0^ 2.1898894 × 10^0^ 2.1886636 × 10^0^ −2.1886636 × 10^0^ 0.0000000 × 10^0^ 0.0000000 × 10^0^ 0.0000000 × 10^0^ 0.0000000 × 10^0^ 0.0000000 × 10^0^ 0.0000000 × 10^0^
[17/18] [17/18] [17/18] [17/18] [17/18] [17/18] [17/18] [17/18] [17/18] [17/18] [17/18] [17/18] [17/18] [17/18] [17/18] [17/18] [17/18] [17/18]	−1.8613610 × 10^2^ −8.1919803 × 10^1^ −3.8053205 × 10^0^ −3.8053205 × 10^0^ 4.3311772 × 10^−2^ 4.3311772 × 10^−2^ 1.1180539 × 10^1^ −2.5486699 × 10^−1^ −2.5486699 × 10^−1^ −1.3851621 × 10^−2^ −1.3851621 × 10^−2^ **−6.0255523 × 10^−2^** **−4.0772993 × 10^−2^** **−4.0088550 × 10^−3^** 1.0171588 × 10^0^ 2.9535681 × 10^0^ 2.9535681 × 10^0^ 3.0011943 × 10^0^	0.0000000 × 10^0^ 0.0000000 × 10^0^ 1.8744806 × 10^1^ −1.8744806 × 10^1^ 1.2476304 × 10^1^ −1.2476304 × 10^1^ 0.0000000 × 10^0^ 6.4629459 × 10^0^ −6.4629459 × 10^0^ 6.2849665 × 10^0^ −6.2849665 × 10^0^ 0.0000000 × 10^0^ 0.0000000 × 10^0^ 0.0000000 × 10^0^ 0.0000000 × 10^0^ 1.0840509 × 10^−1^ −1.0840509 × 10^−1^ 0.0000000 × 10^0^	6.2585091 × 10^4^ −2.5169979 × 10^4^ 3.0764988 × 10^2^ 3.0764988 × 10^2^ 1.7795390 × 10^2^ 1.7795390 × 10^2^ −1.2838628 × 10^−6^ 2.8160877 × 10^−1^ 2.8160877 × 10^−1^ 1.9866901 × 10^2^ 1.9866901 × 10^2^ **2.9023392** **× 10^1^** **1.9842716** **× 10^1^** **1.5017946** **× 10^2^** 3.0059349 × 10^−13^ −1.1383378 × 10^−13^ −1.1383378 × 10^−13^ −1.0771318 × 10^−13^	0.0000000 × 10^0^ 0.0000000 × 10^0^ 3.3948578 × 10^2^ −3.3948578 × 10^2^ 2.2826081 × 10^0^ −2.2826081 × 10^0^ 0.0000000 × 10^0^ 2.1566794 × 10^0^ −2.1566794 × 10^0^ −2.0693506 × 10^0^ 2.0693506 × 10^0^ 0.0000000 × 10^0^ 0.0000000 × 10^0^ 0.0000000 × 10^0^ 0.0000000 × 10^0^ 1.3943289 × 10^−14^ −1.3943289 × 10^−14^ 0.0000000 × 10^0^

**Table 10:** Deconvolution of two-component function $f(t)=25e^{-0.05t}+e^{-0.002t}$ using RPL when $p_{0}=0.1$.

Level	Re(beta)	Im(beta)	Re(alpha)	Im(alpha)
[0/1]	−4.6310442 × 10^−2^	0.0000000 × 10^0^	2.5831230 × 10^1^	0.0000000 × 10^0^
[1/2] [1/2]	−5.3432749 × 10^−2^ −1.5461139 × 10^−2^	0.0000000 × 10^0^ 0.0000000 × 10^0^	2.3078996 × 10^1^ 3.0173646 × 10^0^	0.0000000 × 10^0^ 0.0000000 × 10^0^
[2/3] [2/3] [2/3]	7.5235319 × 10^−1^ −4.9867328 × 10^−2^ −1.6476801 × 10^−3^	0.0000000 × 10^0^ 0.0000000 × 10^0^ 0.0000000 × 10^0^	−1.1384877 × 10^−1^ 2.5009292 × 10^1^ 9.6566322 × 10^−1^	0.0000000 × 10^0^ 0.0000000 × 10^0^ 0.0000000 × 10^0^
[3/4] [3/4] [3/4] [3/4]	−4.8573755 × 10^−2^ −4.8573755 × 10^−2^ **−5.0000000** **× 10^−2^** **−2.0000000** **× 10^−3^**	1.5501904 × 10^1^ −1.5501904 × 10^1^ 0.0000000 × 10^0^ 0.0000000 × 10^0^	6.5083699 × 10^1^ 6.5083699 × 10^1^ **2.5000000** **× 10^1^** **1.0000000** **× 10^0^**	1.7638061 × 10^−3^ −1.7638061 × 10^−3^ 0.0000000 × 10^0^ 0.0000000 × 10^0^
[4/5] [4/5] [4/5] [4/5] [4/5]	−7.3878465 × 10^−2^ −7.3878465 × 10^−2^ 5.7111411 × 10^0^ **−5.0000000** **× 10^−2^** **−2.0000000** **× 10^−3^**	1.5415245 × 10^1^ −1.5415245 × 10^1^ 0.0000000 × 10^0^ 0.0000000 × 10^0^ 0.0000000 × 10^0^	6.4287423 × 10^1^ 6.4287423 × 10^1^ −1.9725482 × 10^−2^ **2.5000000** **× 10^1^** **1.0000000** **× 10^0^**	1.3368022 × 10^−1^ −1.3368022 × 10^−1^ 0.0000000 × 10^0^ 0.0000000 × 10^0^ 0.0000000 × 10^0^
[5/6] [5/6] [5/6] [5/6] [5/6] [5/6]	−4.8802383 × 10^−2^ −4.8802383 × 10^−2^ 1.2369563 × 10^0^ −5.0388491 × 10^−2^ **−4.9999999** **× 10^−2^** **−2.0000000** **× 10^−3^**	1.5484391 × 10^1^ −1.5484391 × 10^1^ 0.0000000 × 10^0^ 0.0000000 × 10^0^ 0.0000000 × 10^0^ 0.0000000 × 10^0^	6.4936263 × 10^1^ 6.4936263 × 10^1^ −5.6204172 × 10^−6^ 5.0487277 × 10^−5^ **2.4999948** **× 10^1^** **1.0000000** **× 10^0^**	2.7538122 × 10^−3^ −2.7538122 × 10^−3^ 0.0000000 × 10^0^ 0.0000000 × 10^0^ 0.0000000 × 10^0^ 0.0000000 × 10^0^
[6/7] [6/7] [6/7] [6/7] [6/7] [6/7] [6/7]	−4.9203724 × 10^−2^ −4.9203724 × 10^−2^ 1.4834847 × 10^0^ −1.5036091 × 10^−2^ −1.5036091 × 10^−2^ **−5.0000000** **× 10^−2^** **−2.0000000** **× 10^−3^**	1.5479164 × 10^1^ −1.5479164 × 10^1^ 0.0000000 × 10^0^ 6.5817021 × 10^−2^ −6.5817021 × 10^−2^ 0.0000000 × 10^0^ 0.0000000 × 10^0^	6.4891961 × 10^1^ 6.4891961 × 10^1^ −1.6903900 × 10^−5^ 6.0655888 × 10^−11^ 6.0655888 × 10^−11^ **2.5000000** **× 10^1^** **1.0000000** **× 10^0^**	4.4870559 × 10^−3^ −4.4870559 × 10^−3^ 0.0000000 × 10^0^ 1.2683360 × 10^−10^ −1.2683360 × 10^−10^ 0.0000000 × 10^0^ 0.0000000 × 10^0^

**Table 11:** Deconvolution of five-component function $f(t)=8560e^{-0.5t}+5650e^{-0.2t}+3725e^{-0.1t}+2358e^{-0.7t}+1350e^{-0.01t}$ using RPL when $p_{0}=1$.

Level	Re(beta)	Im(beta)	Re(alpha)	Im(alpha)
[0/1]	−2.8012545 × 10^−1^	0.0000000 × 10^0^	2.1185167 × 10^4^	0.0000000 × 10^0^
[1/2] [1/2]	−7.9820851 × 10^−1^ −1.6193872 × 10^−1^	0.0000000 × 10^0^ 0.0000000 × 10^0^	7.7648726 × 10^3^ 1.4211874 × 10^4^	7.7648726 × 10^3^ 1.4211874 × 10^4^
[2/3] [2/3] [2/3]	1.0900893 × 10^1^ −4.7531904 × 10^−1^ −8.8258329 × 10^−2^	0.0000000 × 10^0^ 0.0000000 × 10^0^ 0.0000000 × 10^0^	−7.5260406 × 10^2^ 1.3473832 × 10^4^ 7.9883055 × 10^3^	0.0000000 × 10^0^ 0.0000000 × 10^0^ 0.0000000 × 10^0^
[3/4] [3/4] [3/4] [3/4]	9.5438875 × 10^0^ −4.8570632 × 10^−1^ −9.3996711 × 10^−2^ 3.1160963 × 10^−1^	0.0000000 × 10^0^ 0.0000000 × 10^0^ 0.0000000 × 10^0^ 0.0000000 × 10^0^	−5.9804862 × 10^2^ 1.3123770 × 10^4^ 8.3615769 × 10^3^ 1.9220357 × 10^0^	0.0000000 × 10^0^ 0.0000000 × 10^0^ 0.0000000 × 10^0^ 0.0000000 × 10^0^
[4/5] [4/5] [4/5] [4/5] [4/5]	5.9042779 × 10^0^ 5.9042779 × 10^0^ −5.5327113 × 10^−1^ −1.8322055 × 10^−1^ −2.9933826 × 10^−2^	1.6583941 × 10^1^ −1.6583941 × 10^1^ 0.0000000 × 10^0^ 0.0000000 × 10^0^ 0.0000000 × 10^0^	2.1860469 × 10^3^ 2.1860469 × 10^3^ 1.0385843 × 10^4^ 8.5114077 × 10^3^ 2.7148235 × 10^3^	−9.4864888 × 10^2^ 9.4864888 × 10^2^ 0.0000000 × 10^0^ 0.0000000 × 10^0^ 0.0000000 × 10^0^
[5/6] [5/6] [5/6] [5/6] [5/6] [5/6]	2.2977538 × 10^0^ 2.2977538 × 10^0^ −6.1888009 × 10^−1^ −4.0317904 × 10^−1^ −1.4563794 × 10^−1^ −1.6809887 × 10^−2^	6.3404844 × 10^1^ −6.3404844 × 10^1^ 0.0000000 × 10^0^ 0.0000000 × 10^0^ 0.0000000 × 10^0^ 0.0000000 × 10^0^	3.5971774 × 10^4^ 3.5971774 × 10^4^ 6.3141033 × 10^3^ 5.9648905 × 10^3^ 7.5396158 × 10^3^ 1.8221989 × 10^3^	−1.5224157 × 10^3^ 1.5224157 × 10^3^ 0.0000000 × 10^0^ 0.0000000 × 10^0^ 0.0000000 × 10^0^ 0.0000000 × 10^0^
[6/7] [6/7] [6/7] [6/7] [6/7] [6/7] [6/7]	−4.1810290 × 10^−1^ −4.1810290 × 10^−1^ **−7.0161494 × 10^−1^** **−5.0095620 × 10^−1^** **−2.0110761 × 10^−1^** **−1.0085442 × 10^−1^** **−1.0099624 × 10^−2^**	7.7962332 × 10^1^ −7.7962332 × 10^1^ 0.0000000 × 10^0^ 0.0000000 × 10^0^ 0.0000000 × 10^0^ 0.0000000 × 10^0^ 0.0000000 × 10^0^	5.4818688 × 10^4^ 5.4818688 × 10^4^ **2.3126253 × 10^3^** **8.5888020 × 10^3^** **5.5989555 × 10^3^** **3.7854835 × 10^3^** **1.3572038 × 10^3^**	5.2449314 × 10^1^ −5.2449314 × 10^1^ 0.0000000 × 10^0^ 0.0000000 × 10^0^ 0.0000000 × 10^0^ 0.0000000 × 10^0^ 0.0000000 × 10^0^
[7/8] [7/8] [7/8] [7/8] [7/8] [7/8] [7/8] [7/8]	−4.5025997 × 10^−1^ −4.5025997 × 10^−1^ 2.1128167 × 10^1^ **−6.9998756 × 10^−1^** **−4.9999418 × 10^−1^** **−1.9999568 × 10^−1^** **−9.9997141 × 10^−2^** **−9.9997170 × 10^−3^**	7.6911645 × 10^1^ −7.6911645 × 10^1^ 0.0000000 × 10^0^ 0.0000000 × 10^0^ 0.0000000 × 10^0^ 0.0000000 × 10^0^ 0.0000000 × 10^0^ 0.0000000 × 10^0^	5.3301980 × 10^4^ 5.3301980 × 10^4^ −6.3893270 × 10^0^ **2.3583179 × 10^3^** **8.5597566 × 10^3^** **5.6501371 × 10^3^** **3.7247848 × 10^3^** **1.3499779 × 10^3^**	8.5024460 × 10^1^ −8.5024460 × 10^1^ 0.0000000 × 10^0^ 0.0000000 × 10^0^ 0.0000000 × 10^0^ 0.0000000 × 10^0^ 0.0000000 × 10^0^ 0.0000000 × 10^0^

**Table 12:** Deconvolution of noisy data, generated after adding a white Gaussian noise (SNR = 10) to the two-component function $f(t)=25e^{-0.05t}+e^{-0.002t}$, using RPL when $p_{0}=0.1$.

Level	Re(beta)	Im(beta)	Re(alpha)	Im(alpha)
[0/1]	−4.6532813 × 10^−2^	0.0000000 × 10^0^	2.6046383 × 10^1^	0.0000000 × 10^0^
[1/2] [1/2]	−4.9493508 × 10^−2^ 4.3303640 × 10^−3^	0.0000000 × 10^0^ 0.0000000 × 10^0^	2.5618333 × 10^1^ 6.1072333 × 10^−1^	0.0000000 × 10^0^ 0.0000000 × 10^0^
[2/3] [2/3] [2/3]	−2.4527859 × 10^−1^ −5.2343439 × 10^−2^ −7.3617836 × 10^−3^	0.0000000 × 10^0^ 0.0000000 × 10^0^ 0.0000000 × 10^0^	−4.1120854 × 10^−1^ 2.4939697 × 10^1^ 1.6356657 × 10^0^	0.0000000 × 10^0^ 0.0000000 × 10^0^ 0.0000000 × 10^0^
[3/4] [3/4] [3/4] [3/4]	9.2095511 × 10^−2^ 9.2095511 × 10^−2^ **−5.0339820 × 10^−2^** **−2.5538760 × 10^−3^**	1.4129833 × 10^−1^ −1.4129833 × 10^−1^ 0.0000000 × 10^0^ 0.0000000 × 10^0^	−1.8488909 × 10^−3^ −1.8488909 × 10^−3^ **2.5216832 × 10^1^** **1.0215389 × 10^0^**	−4.2125394 × 10^−3^ 4.2125394 × 10^−3^ 0.0000000 × 10^0^ 0.0000000 × 10^0^
[4/5] [4/5] [4/5] [4/5] [4/5]	3.4435350 × 10^−1^ 3.3736775 × 10^−2^ 3.3736775 × 10^−2^ **−4.9906591 × 10^−2^** **−1.5734160 × 10^−3^**	0.0000000 × 10^0^ 1.6601193 × 10^−1^ −1.6601193 × 10^−1^ 0.0000000 × 10^0^ 0.0000000 × 10^0^	−3.5226274 × 10^−3^ 9.1900664 × 10^−3^ 9.1900664 × 10^−3^ **2.5247336 × 10^1^** **9.1880906 × 10^−1^**	0.0000000 × 10^0^ −2.2368378 × 10^−2^ 2.2368378 × 10^−2^ 0.0000000 × 10^0^ 0.0000000 × 10^0^
[5/6] [5/6] [5/6] [5/6] [5/6] [5/6]	1.9989881 × 10^−1^ 1.9989881 × 10^−1^ −4.9507620 × 10^−3^ −4.9507620 × 10^−3^ **−5.0495725 × 10^−2^** **−2.1235336 × 10^−3^**	4.7267992 × 10^−1^ −4.7267992 × 10^−1^ 1.1479799 × 10^−1^ −1.1479799 × 10^−1^ 0.0000000 × 10^0^ 0.0000000 × 10^0^	6.2996699 × 10^−2^ 6.2996699 × 10^−2^ 1.1328981 × 10^−2^ 1.1328981 × 10^−2^ **2.5398527 × 10^1^** **9.8862834 × 10^−1^**	9.9681731 × 10^−2^ −9.9681731 × 10^−2^ 3.5395658 × 10^−2^ −3.5395658 × 10^−2^ 0.0000000 × 10^0^ 0.0000000 × 10^0^
[6/7] [6/7] [6/7] [6/7] [6/7] [6/7] [6/7]	−4.7121082 × 10^−1^ −4.7121082 × 10^−1^ −9.8173414 × 10^−2^ −1.6574766 × 10^−2^ −1.6574766 × 10^−2^ −4.6842552 × 10^−2^ −1.4529226 × 10^−3^	8.8269035 × 10^−1^ −8.8269035 × 10^−1^ 0.0000000 × 10^0^ 7.8229861 × 10^−2^ −7.8229861 × 10^−2^ 0.0000000 × 10^0^ 0.0000000 × 10^0^	8.3216962 × 10^−1^ 8.3216962 × 10^−1^ 4.7499294 × 10^0^ −6.7687562 × 10^−2^ −6.7687562 × 10^−2^ 2.1736778 × 10^1^ 8.7883033 × 10^−1^	2.1437565 × 10^0^ −2.1437565 × 10^0^ 0.0000000 × 10^0^ −5.7532139 × 10^−2^ 5.7532139 × 10^−2^ 0.0000000 × 10^0^ 0.0000000 × 10^0^

**Table 13:** Deconvolution of three-component function $f(t)=40e^{-0.002t}+35e^{-0.009t}-60e^{-0.05t},$ using RPL when $p_{0}=0.05$.

Level	Re(beta)	Im(beta)	Re(alpha)	Im(alpha)
[0/1]	9.5517727 × 10^−3^	0.0000000 × 10^0^	3.0833546 × 10^1^	0.0000000 × 10^0^
[1/2] [1/2]	−5.3318240 × 10^−2^ −3.9710375 × 10^−3^	0.0000000 × 10^0^ 0.0000000 × 10^0^	−5.5129353 × 10^1^ 6.9940225 × 10^1^	0.0000000 × 10^0^ 0.0000000 × 10^0^
[2/3] [2/3] [2/3]	−4.8722175 × 10^−2^ −1.4227833 × 10^−2^ −2.8106907 × 10^−3^	0.0000000 × 10^0^ 0.0000000 × 10^0^ 0.0000000 × 10^0^	−6.3175619 × 10^1^ 2.3348338 × 10^1^ 5.4854799 × 10^1^	0.0000000 × 10^0^ 0.0000000 × 10^0^ 0.0000000 × 10^0^
[3/4] [3/4] [3/4] [3/4]	3.4837912 × 10^−1^ −5.0134633 × 10^−2^ −8.8230772 × 10^−3^ −1.9566787 × 10^−3^	0.0000000 × 10^0^ 0.0000000 × 10^0^ 0.0000000 × 10^0^ 0.0000000 × 10^0^	−3.1650844 × 10^−2^ −5.9897040 × 10^1^ 3.5668241 × 10^1^ 3.9174859 × 10^1^	0.0000000 × 10^0^ 0.0000000 × 10^0^ 0.0000000 × 10^0^ 0.0000000 × 10^0^
[4/5] [4/5] [4/5] [4/5] [4/5]	1.7194367 × 10^−1^ 1.7194367 × 10^−1^ **−5.0000000 × 10^−2^** **−9.0000000 × 10^−3^** **−2.0000000 × 10^−3^**	7.7165657 × 10^0^ −7.7165657 × 10^0^ 0.0000000 × 10^0^ 0.0000000 × 10^0^ 0.0000000 × 10^0^	3.7289858 × 10^1^ 3.7289858 × 10^1^ **−6.0000000 × 10^1^** **3.5000000 × 10^1^** **4.0000000 × 10^1^**	7.0358919 × 10^−3^ −7.0358919 × 10^−3^ 0.0000000 × 10^0^ 0.0000000 × 10^0^ 0.0000000 × 10^0^
[5/6] [5/6] [5/6] [5/6] [5/6] [5/6]	1.7196744 × 10^−1^ 1.7196744 × 10^−1^ **−5.0000000** **× 10^−2^** −4.1165123 × 10^−2^ **−9.0000000** **× 10^−3^** **−2.0000000** **× 10^−3^**	7.7153057 × 10^0^ −7.7153057 × 10^0^ 0.0000000 × 10^0^ 0.0000000 × 10^0^ 0.0000000 × 10^0^ 0.0000000 × 10^0^	3.7277667 × 10^1^ 3.7277667 × 10^1^ **−5.9999998** **× 10^1^** −2.1944340 × 10^−6^ **3.5000000** **× 10^1^** **4.0000000** **× 10^1^**	6.9193200 × 10^−3^ −6.9193200 × 10^−3^ 0.0000000 × 10^0^ 0.0000000 × 10^0^ 0.0000000 × 10^0^ 0.0000000 × 10^0^
[6/7] [6/7] [6/7] [6/7] [6/7] [6/7] [6/7]	1.7193555 × 10^−1^ 1.7193555 × 10^−1^ 7.6658423 × 10^−3^ 7.6658423 × 10^−3^ **−5.0000000** **× 10^−2^** **−9.0000000** **× 10^−3^** **−2.0000000** **× 10^−3^**	7.7163519 × 10^0^ −7.7163519 × 10^0^ 6.8951426 × 10^−2^ −6.8951426 × 10^−2^ 0.0000000 × 10^0^ 0.0000000 × 10^0^ 0.0000000 × 10^0^	3.7287794 × 10^1^ 3.7287794 × 10^1^ −7.5593396 × 10^−11^ −7.5593396 × 10^−11^ **−6.0000000** **× 10^1^** **3.5000000** **× 10^1^** **4.0000000** **× 10^1^**	7.0750599 × 10^−3^ −7.0750599 × 10^−3^ −1.3222430 × 10^−11^ 1.3222430 × 10^−11^ 0.0000000 × 10^0^ 0.0000000 × 10^0^ 0.0000000 × 10^0^
[7/8] [7/8] [7/8] [7/8] [7/8] [7/8] [7/8] [7/8]	1.7194602 × 10^−1^ 1.7194602 × 10^−1^ 9.3410071 × 10^−2^ **−5.0000000** **× 10^−2^** 1.9105494 × 10^−3^ 1.9105494 × 10^−3^ **−9.0000000** **× 10^−3^** **−2.0000000** **× 10^−3^**	7.7159127 × 10^0^ −7.7159127 × 10^0^ 0.0000000 × 10^0^ 0.0000000 × 10^0^ 5.3649894 × 10^−2^ −5.3649894 × 10^−2^ 0.0000000 × 10^0^ 0.0000000 × 10^0^	3.7283544 × 10^1^ 3.7283544 × 10^1^ −4.8135249 × 10^−11^ **−6.0000000** **× 10^1^** 3.6900374 × 10^−10^ 3.6900374 × 10^−10^ **3.5000000** **× 10^1^** **4.0000000** **× 10^1^**	7.0238724 × 10^−3^ −7.0238724 × 10^−3^ 0.0000000 × 10^0^ 0.0000000 × 10^0^ −2.1846720 × 10^−10^ 2.1846720 × 10^−10^ 0.0000000 × 10^0^ 0.0000000 × 10^0^

**Table 14:** Deconvolution of noisy data, generated after adding a white Gaussian noise (SNR = 10) to the function $f(t)=40e^{-0.002t}+35e^{-0.009t}-60e^{-0.05t},$ using RPL when $p_{0}=0.05$.

Level	Re(beta)	Im(beta)	Re(alpha)	Im(alpha)
[3/4] [3/4] [3/4] [3/4]	−1.6916657 × 10^−1^ −5.2380624 × 10^−2^ −6.6573511 × 10^−3^ −1.2001808 × 10^−3^	0.0000000 × 10^0^ 0.0000000 × 10^0^ 0.0000000 × 10^0^ 0.0000000 × 10^0^	4.4097682 × 10^−1^ −5.8372837 × 10^1^ 4.6990984 × 10^1^ 2.5637257 × 10^1^	0.0000000 × 10^0^ 0.0000000 × 10^0^ 0.0000000 × 10^0^ 0.0000000 × 10^0^
[4/5] [4/5] [4/5] [4/5] [4/5]	−1.4839124 × 10^−1^ −5.2498616 × 10^−2^ −6.5669995 × 10^−3^ −1.1390847 × 10^−3^ 6.3259167 × 10^−2^	0.0000000 × 10^0^ 0.0000000 × 10^0^ 0.0000000 × 10^0^ 0.0000000 × 10^0^ 0.0000000 × 10^0^	5.5301494 × 10^−1^ −5.8412725 × 10^1^ 4.7777205 × 10^1^ 2.4774822 × 10^1^ −4.0520354 × 10^−10^	0.0000000 × 10^0^ 0.0000000 × 10^0^ 0.0000000 × 10^0^ 0.0000000 × 10^0^ 0.0000000 × 10^0^
[5/6] [5/6] [5/6] [5/6] [5/6] [5/6]	1.5252952 × 10^−1^ −5.1367483 × 10^−2^ 1.4373202 × 10^−2^ 1.4373202 × 10^−2^ −7.8249136 × 10^−3^ −1.7322604 × 10^−3^	0.0000000 × 10^0^ 0.0000000 × 10^0^ 4.8906437 × 10^−2^ −4.8906437 × 10^−2^ 0.0000000 × 10^0^ 0.0000000 × 10^0^	−3.7967646 × 10^−4^ −5.9007827 × 10^1^ 2.8204331 × 10^−3^ 2.8204331 × 10^−3^ 3.9143448 × 10^1^ 3.4508292 × 10^1^	0.0000000 × 10^0^ 0.0000000 × 10^0^ −1.6883927 × 10^−3^ 1.6883927 × 10^−3^ 0.0000000 × 10^0^ 0.0000000 × 10^0^
[6/7] [6/7] [6/7] [6/7] [6/7] [6/7] [6/7]	1.0670606 × 10^−1^ 1.0670606 × 10^−1^ −2.6417235 × 10^−2^ −2.6417235 × 10^−2^ **−4.8651982** **× 10^−2^** **−9.2794132** **× 10^−3^** **−2.0087154** **× 10^−3^**	1.6000709 × 10^−1^ −1.6000709 × 10^−1^ 5.3662647 × 10^−2^ −5.3662647 × 10^−2^ 0.0000000 × 10^0^ 0.0000000 × 10^0^ 0.0000000 × 10^0^	−8.9423565 × 10^−3^ −8.9423565 × 10^−3^ 8.8029576 × 10^−2^ 8.8029576 × 10^−2^ **−6.1368344** **× 10^1^** **3.5660235** **× 10^1^** **4.0347352** **× 10^1^**	1.2589839 × 10^−2^ −1.2589839 × 10^−2^ −4.2733350 × 10^−1^ 4.2733350 × 10^−1^ 0.0000000 × 10^0^ 0.0000000 × 10^0^ 0.0000000 × 10^0^
[7/8] [7/8] [7/8] [7/8] [7/8] [7/8] [7/8] [7/8]	3.2939684 × 10^−1^ 4.4906532 × 10^−2^ 4.4906532 × 10^−2^ **−5.0840652** **× 10^−2^** −1.2343101 × 10^−2^ −1.2343101 × 10^−2^ **−8.8362213** **× 10^−3^** **−1.9621520** **× 10^−3^**	0.0000000 × 10^0^ 1.1239422 × 10^−1^ −1.1239422 × 10^−1^ 0.0000000 × 10^0^ 4.0179838 × 10^−2^ −4.0179838 × 10^−2^ 0.0000000 × 10^0^ 0.0000000 × 10^0^	−1.9983042 × 10^−2^ 4.4950812 × 10^−3^ 4.4950812 × 10^−3^ **−6.0463730** **× 10^1^** 1.0214574 × 10^−1^ 1.0214574 × 10^−1^ **3.5620994** **× 10^1^** **3.9226211** **× 10^1^**	0.0000000 × 10^0^ −4.1878055 × 10^−3^ 4.1878055 × 10^−3^ 0.0000000 × 10^0^ 8.3652044 × 10^−2^ −8.3652044 × 10^−2^ 0.0000000 × 10^0^ 0.0000000 × 10^0^
[8/9] [8/9] [8/9] [8/9] [8/9] [8/9] [8/9] [8/9] [8/9]	8.7551404 × 10^−1^ 4.8439223 × 10^−3^ 4.8439223 × 10^−3^ −6.2372719 × 10^−2^ −3.7786151 × 10^−2^ 1.1713447 × 10^−2^ 1.1713447 × 10^−2^ −9.5422085 × 10^−3^ −2.0198601 × 10^−3^	0.0000000 × 10^0^ 1.2261909 × 10^−1^ −1.2261909 × 10^−1^ 0.0000000 × 10^0^ 0.0000000 × 10^0^ 3.6263972 × 10^−2^ −3.6263972 × 10^−2^ 0.0000000 × 10^0^ 0.0000000 × 10^0^	−4.1265290 × 10^−1^ 6.7327908 × 10^−2^ 6.7327908 × 10^−2^ −3.3812097 × 10^1^ −2.9331610 × 10^1^ 8.1928119 × 10^−5^ 8.1928119 × 10^−5^ 3.6568214 × 10^1^ 4.0687095 × 10^1^	0.0000000 × 10^0^ 1.1109084 × 10^−2^ −1.1109084 × 10^−2^ 0.0000000 × 10^0^ 0.0000000 × 10^0^ 3.9570379 × 10^−4^ −3.9570379 × 10^−4^ 0.0000000 × 10^0^ 0.0000000 × 10^0^

## Data Availability

Not applicable.

## Appendix A

(A1) $$\begin{array}{l} \frac{a_{0}+a_{1}(p-p_{0})+a_{2}{\left(p-p_{0}\right)}^{2}+\cdots +a_{n-1}{\left(p-p_{0}\right)}^{n-1}}{1+b_{1}(p-p_{0})+b_{2}{\left(p-p_{0}\right)}^{2}+\cdots +b_{n}{\left(p-p_{0}\right)}^{n}}=\\ c_{0}+c_{1}(p-p_{0})+c_{2}{\left(p-p_{0}\right)}^{2}+\cdots +c_{2n-1}{\left(p-p_{0}\right)}^{2n-1} \end{array}$$

(A2) $$\begin{array}{c} a_{0}+a_{1}\left(p-p_{0}\right)+a_{2}{\left(p-p_{0}\right)}^{2}+\cdots +a_{n-1}{\left(p-p_{0}\right)}^{n-1}=\\ c_{0}+c_{1}\left(p-p_{0}\right)+c_{2}{\left(p-p_{0}\right)}^{2}+\cdots +c_{2n-1}{\left(p-p_{0}\right)}^{2n-1}+\\ b_{1}c_{0}\left(p-p_{0}\right)+b_{1}c_{1}{\left(p-p_{0}\right)}^{2}+b_{1}c_{2}{\left(p-p_{0}\right)}^{3}+\cdots +b_{1}c_{2n-2}{\left(p-p_{0}\right)}^{2n-1}+\cdots +\\ b_{2}c_{0}{\left(p-p_{0}\right)}^{2}+b_{2}c_{1}{\left(p-p_{0}\right)}^{3}+b_{2}c_{2}{\left(p-p_{0}\right)}^{4}+\cdots +b_{2}c_{2n-3}{\left(p-p_{0}\right)}^{2n-1}+\cdots +\\ b_{n}c_{0}{\left(p-p_{0}\right)}^{n}+b_{n}c_{1}{\left(p-p_{0}\right)}^{n+1}+b_{n}c_{2}{\left(p-p_{0}\right)}^{n+2}+\cdots +b_{n}c_{n-1}{\left(p-p_{0}\right)}^{2n-1}+\cdots \end{array}$$

(A3) $$\begin{array}{l} a_{0}=c_{0}\\ a_{1}=c_{1}+b_{1}c_{0}\\ a_{2}=c_{2}+b_{1}c_{1}+b_{2}c_{0}\\ a_{3}=c_{3}+b_{1}c_{2}+b_{2}c_{1}+b_{3}c_{0}\\ \vdots \\ a_{n-1}=c_{n-1}+b_{1}c_{n-2}+b_{2}c_{n-3}+b_{3}c_{n-4}+\cdots +b_{n-1}c_{0} \end{array}$$

(A4) $$\begin{array}{ll} a_{0}=c_{0},\; & \\ a_{k}=c_{k}+\overset{k}{\underset{i=1}{\sum }}b_{i}c_{k-i}, & 0<k\leq n-1 \end{array}$$

(A5) $$\begin{array}{l} c_{n}+b_{1}c_{n-1}+b_{2}c_{n-2}+\cdots +b_{n}c_{0}=0\\ c_{n+1}+b_{1}c_{n}+b_{2}c_{n-1}+\cdots +b_{n}c_{1}=0\\ c_{n+2}+b_{1}c_{n+1}+b_{2}c_{n}+\cdots +b_{n}c_{2}=0\\ \vdots \\ c_{2n-1}+b_{1}c_{2n-2}+b_{2}c_{2n-3}+\cdots +b_{n}c_{n-1}=0 \end{array}$$

(A6) $$c_{k}+\overset{n}{\underset{i=1}{\sum }}b_{i}c_{k-i}=0,\;\;n\leq k\leq 2n-1$$
